# Numbers don’t speak for themselves: strategies of using numbers in public policy discourse

**DOI:** 10.1007/s10649-021-10059-8

**Published:** 2021-05-12

**Authors:** Eva Jablonka, Christer Bergsten

**Affiliations:** 1 Department of Education and Psychology, Freie Universität Berlin, Berlin, Germany; 2 Department of Mathematics, Linköpings universitet, Linköping, Sweden

**Keywords:** Mathematical literacy, Critical mathematics education, Mathematisation, Policy discourse, Discursive strategies, COVID-19, Post-structuralist discourse theory

## Abstract

In mathematics education, there is general agreement regarding the significance of mathematical literacy (also quantitative literacy or numeracy) for informed citizenship, which often requires evaluating the use of numbers in public policy discourse. We hold that such an evaluation must accommodate the necessarily fragile relation between the information that numbers are taken to carry and the policy decisions they are meant to support. In doing so, attention needs to be paid to differences in *how* that relation is formed. With this in mind, we investigated a public discourse that heavily relied on numbers in the context of introducing, maintaining, and easing the rules and regulations directed to contain the spread of the virus SARS-CoV-2 during the first epidemic wave of COVID-19 in Germany with its peak in early April 2020. We used a public-service broadcasting outlet as data. Our theoretical stance is affiliated with post-structuralist discourse theory. As an outcome, we identified four major related strategies of using numbers, which we named *rationalisation*, *contrast*, *association* and *recharging*. In our view explicit attention to these strategies as well as identifying new ones can aid the task of furthering critical mathematical literacy.

## Background and aims

Goal statements of mathematics education routinely emphasise the significance of mathematical literacy (also quantitative literacy or numeracy) for informed critical citizens. These goals comprise understanding the use of mathematics in public discourse, in particular concerning controversial policies, including the critical evaluation of the very role mathematics plays in these discourses (Geiger et al., 2015; Jablonka, 2015; Niss & Jablonka, 2020; Skovsmose, 2021). As to public discourses in which numbers feature prominently, important contributions furthering mathematical and statistical literacy concern identifying common pitfalls in interpretations of central concepts (e.g., Bakker et al., 2004; Pierce & Chick, 2013; Pratt et al., 2011; Van Dooren et al., 2003), scrutinising the process of measurement and modelling along with attention to what has been omitted in the quantification (e.g., Barbosa, 2006; Jablonka, 1997) as well as excavating the invisible mathematical models from which numbers are emitted into the public sphere (e.g., Gellert & Jablonka, 2007; Iceland, 2005; Keitel et al., 1993). In the context of the COVID-19 pandemic, some of these educational tasks have even been adopted by the prestigious press (e.g., Paulos, 2020; Richardson & Spiegelhalter, 2020).

However, a critical evaluation of the use of numbers by political actors would be naïve, if it were confined to determining whether “numbers are used wrongly” and/or “the wrong numbers are used”. While this appears possible in familiar contexts by habit, or in the face of complex problems by agreement on scientific and moral standards for the construction and use of mathematical models and statistics, such a judgement reaches at all events beyond mathematical literacy conceived as a *mathematical* competence. Political dimensions of the use of numbers have been explicitly recognised in the tradition of critical mathematical and statistical literacy. These concern both (i) unmasking of the apparent neutrality of depicting the social sphere by means of quantities, metrics or indicators, and (ii) analysing the selection and adaptation of numbers in the service of particular interests (e.g., Appelbaum & Davila, 2009; Brantlinger, 2014; Fish & Persaud, 2012; Frankenstein, 2008; Jablonka, 1997; Skovsmose, 1992; Weiland, 2017). The second of these goals has become mainstreamed in conceptions of statistical and probability literacy (e.g., Gal, 2005; Shaughnessy, 2007; Watson, 1997; Watson & Callingham, 2020). However, it is not a straightforward exercise. Numbers don’t speak for themselves.

Some more specific questions that have been addressed regarding the use of numbers by political actors include revealing inflation and deflation of numbers referring to the same problem in antagonistic discourses (e.g., Hauge & Barwell, 2015; Munter, 1994), deconstructing particular quantifications (e.g., Frankenstein, 2009; Gutstein, 2008; Sánchez & Blomhøj, 2015; Volk, 1987), and unveiling the “ideology of certainty” (Borba & Skovsmose, 1997; Chassapis, 2017; Straehler-Pohl, 2015). Despite that, *how* particular interpretations of numbers in day-to-day political practice are actually achieved has received less attention. In this paper, we examine this question by means of an investigation of the policy discourse in the context of introducing, maintaining and easing the rules and regulations directed to contain the spread of the virus SARS-CoV-2 in Germany during the first wave from January until May 2020. During this period, a dynamic and unfinished construction of the core problem to be solved emerged at each stage of the development. The quantities, metrics or indicators used (henceforth all denoted as “numbers”) were generated in (infectious disease or economic) epidemiology, infectiology, virology, demography and public health care resource statistics; the fields of law, sociology or social psychology played a minor role. Even beyond the topic of expert knowledge, the discourse included the daily production of numbers on liveblogs, interactive maps and graphics. While we are aware of the specificities of this particular context, similar strategies of using numbers as those we identified in our study might be found in other contexts where far-ranging decisions are being made and communicated by politicians and affiliated experts (e.g., environmental degradation, the migration and displacement of people).

A shared vantage point in critical mathematics education concerns the anti-realist or anti-essentialist position regarding mathematical knowledge (Ernest et al., 2016; Kollosche, 2021). In line with this, we assume that the relation between any information numbers are taken to carry and some policy decisions they are meant to support is necessarily fragile. Hence, in the context we analyse, any relation between a specific rule or regulation (e.g., closing schools) with a specific selection or combination of any numbers (e.g., effective reproduction number) is non-necessary and will escape full determination by the experts involved (which by no means implies that the use of numbers is unproductive and that agreement cannot be reached^1^). Starting from these observations and assumptions, the questions we seek to explore are:

- *What are the strategies of using numbers*
  - *in the (changing) constructions of what constitutes the “Corona crisis” (or the absence of a crisis)?*
  - *for establishing, defending or easing rules and regulations and achieving acquiescence?*

Hence, our interest concerns a policy discourse, which heavily relies on the use of numbers, and its embedded strategies of manoeuvring the relation between the numbers, the problem and the policy measures to overcome it, with the explicit goal of achieving compliance. We propose that attention to discursive strategies can aid the task of examining the use of numbers in theoretical and practical work that aims at developing critical mathematical literacy.

## Theoretical horizon and discursive context

In accordance with our aim to explore discursive strategies of using numbers in day-to-day policy, our analysis is inspired by post-structuralist discourse theory. In this account, political identities are shaped by discursive struggles. These aim to establish fixed meanings of particular elements (such as agendas, concepts, behaviours, events, etc.) in relation to key political ideas that promise a full realisation of what is missing (e.g., “order”, “sustainability”) vis-à-vis what hinders this realisation. A discourse becomes “hegemonic” by establishing a (partial and forged) equivalence between those elements and thereby cancelling out differences. In this process, the fixation of meanings might be stabilised and maintained by practices that get a grip on the subject through invoking fantasies and affective investment. (Dahlberg & Phelan, 2011; Glynos, 2001; Howarth, 2010; Laclau, 2007; Laclau & Mouffe, 1985/2001)

Seen from this perspective, the specificity of the discourse we looked at concerns its explicitly intended disciplinary effect and its relatively short-lived articulation, offering only short-term identification. Further, it was articulated from the position of an established regime in the context of a crisis^2^. The stipulations and recommendations for curbing the pandemic came from government bodies and their affiliated experts, majority decisions in parliament and local legislative authorities, sometimes with unexpected political coalitions. Around the key idea of enacting “solidarity” for a collective purpose, the discourse temporarily stabilised the meanings of concepts, events, objects, operations, behaviour etc. that counted as equivalent to “mastering the Corona crisis” (e.g., “social distancing”) as well as demarking what hindered this process (e.g., “private social gatherings”). A concomitant dichotomisation of the social space in adherents and sceptics of the policy measures was observed by Vogel (2020). In line with Phelan and Dahlberg’s (2011, p. 28) observation that media reporting of traumatic events often includes picturing a “unified community” with a tendency to mask social differences within the community, the recognition of unequal conditions for being able to adhere to the measures remained obliterated. Some groups with normally incompatible demands joined in a populist movement that opposed the regulations introduced by the government. While some experts with alternative definitions of the “crisis” and diverging policy suggestions appeared, without much visible conflict these were marginalised. Compliance with the policy measures during the first wave in Germany, in particular in reducing mobility (Schlosser et al., 2020), justifies talking about a successful practice.

As explained in the introduction (Sect. 1), our interest concerns the strategies of using numbers. With *strategies* we here refer to discursive patterns at the micro-level that contributed to the hegemonic status of the discourse within the constellation described above. We do not interpret these strategies as being stylistic forms or rhetorical devices tactically chosen from a variety of options, but also do not (and cannot) exclude that this is the case. Further, we note that from the theoretical perspective outlined above the emergence of the virus constitutes itself as a *specific* object only *within* the discursive articulation (e.g., as natural phenomenon visible through numbers, as expression of fate, or as man-made weapon).

In addition, we assume that political actors in a rational policy discourse that heavily relies on experts from science are not in a position to have a motive for shaking confidence in the trust of the numbers provided, even if in the particular situation the impossibility of separating the process of the spread of the virus from its context (a separation that would be necessary for causal explanations in mathematical models) shines through. Against this background, we interpret the “ideology of certainty” of mathematics (cf. Sect. 1) as an effect of the concealment of its built-in undecidability and of the impossibility of conceptualising its own limits of application.

On that account, we expected that what represents a rational policy discourse in the context of the pandemic will consist in giving evidence by means of numbers and use of argument, while minimising emotive aspects of communication. Yet, from our theoretical vantage point, agreement, acceptance or acquiescence are not only emulated at the level of informed conscious consent; affect, emotion or fantasy contribute to stabilising identifications. Hence, attention to these two aspects guided our exploration of strategies for manoeuvring the relation between the numbers, the problem and the policy measures.

## Methods

### Period of interest

From the end of January until May 2020, the first wave of the COVID-19 pandemic occurred in Germany. In early March, the German federal government banned mass events, mid-March schools were closed before guidelines to limit social contacts were issued and correspondent ordinances and general decrees passed by the state governments. For these measures aimed at curbing the epidemic, the English terms *Lockdown* or *Shutdown* became common. In early May, the federal and state governments decided to gradually ease restrictions; the implementation of *Corona-Lockerungen* (easing of restrictions) began. Then, in the face of an imminent recession, numbers emanating from economics dominated. However, an analysis of the economic policy measures is beyond the scope of this paper, which focusses on the regulative measures concerning the conduct of the people. Hence, we focussed on the period from early January until the end of May.

### Data

For accessing the public policy discourse disseminated by the political centre, we have chosen a public service broadcasting outlet: the website of the news programme *Tagesschau* (“Daily Show”), which offers reports, short presentations and interviews with members of political institutions or experts. The outlet is produced by the German public service television network (ARD), whose free-to-air television-channel has the largest share of comparable news casts (Weidenbach, 2020). The coverage followed the political-administrative processes; shares reached particular high levels when policy measures were announced (Mantel, 2020). Some studies have revealed a systematic bias of the ARD resulting from overrepresenting the German government during the Greek national debt crisis (Otto et al., 2016) and also the COVID-19 pandemic (Hanitzsch, 2020; Jarren, 2020). Altogether, these observations justify our choice to look at this news outlet as representing a public policy discourse from the position of an established regime.

As corpus we used the 365-days archive of the website. As a news item we treated anything assembled under one heading (we did not include blogs and full-length regular television newscasts). There were around 20–50 such items per day. The earliest report about COVID-19 (then “a mysterious lung disease”) appeared on January 6, 2020. In a first step, we looked through 135 randomly selected items out of those 1772 (from a total of 4918) that focussed on the coronavirus in the period from January 6 to May 26. After assembling an overview of the (changing) key metrics used, we chose 40 news items for an in-depth analysis (see Appendix). Criteria for selection included the use of numbers for framing the content and/or the elaboration of at least one theme by means of numbers in the texts and the spread of the examples over the period in order to cover both the establishment and maintenance of the discourse.

During the period of observation there were around 35 news items per day, of which on average about one third focussed on some aspect of the pandemic. However, the number of coronavirus-related items per day remained small till early March when the number of cases in Germany rose. The Corona coverage dominated the reporting from March 12 onwards to the extent that on one single day (March 30) all 26 news items on the website were related to the pandemic.

### Analysis

As a base for the analysis of the selected examples, we assembled detailed thematic summaries of the texts (in the widest sense, including language, images, diagrams, film, animations) alongside their sequential thematic structure, including key literal quotes. This choice was feasible as the structure of the news items was mostly quite simple (e.g., substitutions appeared often directly in consecutive sentences). When journalists summarised speeches, we relied on their summaries only if the original speech was not part of the news item (e.g., via an embedded link to a press conference) or if the speech was not the major topic. When in an item a new theme was entirely unrelated to the major topic, we did not take these passages into account. If passages were not related to either numbers or policy measures, we also omitted these. As to the images, we included all that appeared in the news item because all are visible simultaneously. For each summary we then identified the themes referring to what constitutes the “crisis” and others that referred to concepts, events, objects, operations, behaviour, etc. that related to mastering it.

Motivated by the aims and theoretical horizon of the study, in a first step of the in-depth analysis, we used the thematic summaries as a basis for identifying reference to numbers as elements of scientific discourse in demarcation of other strategies for navigating the relation between the numbers, the crisis and the policy measures to overcome it. As what can be produced (or not) in a scientific discourse is institutionalised, we did not face problems with this. Within these, we inductively found two modes: the use of argument for establishing the relation between numbers that refer to the “reality” of the virus and the envisaged policy measures, and another that does not use argument. We termed these strategies *rationalisation by explanation* and *rationalisation by conjunction*, respectively (see Sect. 4.2.1 and 4.2.2).

In the second step, we assembled all items that did not use rationalisation. Within these, by an inductive process, we identified explicit comparisons of numbers (which we termed *contrast;* see Sect. 4.2.3) or a combination of the use of numbers with a concrete representation of something equivalent with overcoming the crisis or hindering this (*association*; see Sect. 4.2.4), as well as what we termed *recharging* (see Sect. 4.2.5). As we proceeded (in the order of time not to lose track of the overall development of the discourse), we found that the data were saturated as we could not identify other than these overall strategies in the remaining texts; we also did not feel a need for further differentiation within the strategies.

One difficulty of the analysis consisted in the fact that the strategies were mostly combined and used in a nested way, as they are independent of the level of text and so can also be identified on a small scale. However, this turned out not to be a limitation; it rather shows that the strategies are not necessarily bound to the choice of distinct rhetoric devices but in fact are quite flexible. It is certainly possible to provide a more fine-grained analysis, including a depiction of the nested hierarchies of the strategies we found. However, our analysis only aimed to identify major discursive strategies that involve the use of numbers.

## Findings

### The numbers used

Our first observation about the use of numbers concerns the switch of metrics in the discourse with the introduction, maintenance and easing of the rules and regulations as illustrated by the development of frequencies of some key metrics in Table 1. In the beginning, in particular before the number of reported cases of infections rose in Europe, the focus was the mysteriousness of the disease rather than numbers. Initially, only simple metrics were used, including (accumulated or daily) “cases” (of infections, German *Fallzahlen* or *Fälle*) and “deaths” or “fatalities” (*Todesfälle*). We found a variety of labels used for these metrics (e.g., infections, *Infektionen* or *Ansteckungen*). In addition to these simple metrics that later also included “recovered” (*Genesene*), there were ever more complex numbers invoked in the construction of the problem. In the middle of our observation period the metrics “doubling time” (*Verdopplungszeit*) and “case fatality rate” (e.g., *Sterberate*, *Letalitätsrate*, or *Fallsterblichkeit*) appeared. Later, the more complex epidemiological metric “reproduction number” (*Reproduktionszahl*) became frequently used, as well as “excess mortality” (*Übersterblichkeit*), with “doubling time” now appearing less frequently. For the “reproduction number” alternative labels were common (e.g., R-value, *R-Wert*, and infection rate, *Ansteckungsrate*). The use of the (national and regional) “infections per 100000 inhabitants” or “incidence”, accumulated or weekly (*Infektionen pro 100000 Einwohner*, or *Inzidenz*), also increased. Hence, while the use of absolute numbers prevailed, a range of relative numbers became more common when the number of cases rose.^3^

**Table 1:** Frequencies of the most common metrics appearing in our sample of 135 news items

Period (news items)	Cases	New cases	Incidence	Deaths	Case fatality rate	Excess mortality	Re-covered	Doubling time	*R-* value	Total
Jan/Feb (39)	106	14	-	42	3	-	-	-	-	165
March (44)	152	10	4	52	11	-	3	13	1	246
April/May (52)	78	57	15	57	10	10	12	14	125	378
Total (135)	336	81	19	151	24	10	15	27	126	789

These quantities, metrics or indicators were introduced, updated or occasionally “explained” by a cadre of experts with academic leadership functions and taken up by high-level representatives of the national and state governments. Amongst the experts involved regularly, there were the director and vice director of Berlin’s *Robert Koch-Institut* (*RKI*), the government’s central scientific institution for the control and prevention of diseases, and leading virologists at Berlin’s *Charité*.^4^

### Strategies of the use of numbers

We identified four major discursive strategies of suggesting specific interpretations of numbers in (changing) constructions of what constitutes the “Corona crisis” (or the absence of a crisis) and linking these to policy measures. We termed these strategies *rationalisation* (by *explanation* or by *conjunction*), *contrast*, *association and recharging*. In the following we describe and illustrate these by means of examples. For this we use abbreviated versions of the thematic summaries (see method of analysis, Sect. 3.3). An overview of the strategies that we identified in the 40 selected examples is presented in Table 2 (see Appendix).

#### Rationalisation by explanation

This strategy consists in using quantities, metrics or indicators in factual statements for providing evidence, in (conditional) arguments and in explanations, that is, elements of scientific discourse from the relevant fields, as a basis for suggesting policy measures. It so delineates not only what counts as a rational policy but also the behaviour of (not) adhering to the measures as (ir)rational. Further, in a discourse disseminated by government bodies that heavily rely on affiliated experts the status of the authors also appeared important (e.g., professor versus research student or minister of health versus employee of a local health department). The strategy was combined with or shaded off into others, or came with other strategies as sub-strategies. The most prominent examples include official announcements by advisory bodies and explications by experts.

- Example 1: *Coronavirus: keep distance, slow down the virus* (March 14)

The themes of this report are new rules and regulations (“No school, no events, keep your distance: there are drastic measures - and everything has only one goal: to slow the pace of Corona spreading”). Further, there is a description of what “scientists” call “epidemiological curve”. Then, China is mentioned, where “one infected person infected on average two other persons”, a pattern now observed in many European countries where “the number of infections doubles about each three to four days”, which is called “exponential growth and leads quickly to gigantic numbers”. Further, there is a “theoretical calculation example”, stating, “If one assumes a doubling time of 4 days, the number of 1000 infected will increase to about 250,000 after just 1 month. After 2 months, it would be more than 32 million”. It is stated, however, that “epidemiologists” use more complex calculations.

An image entitled “Containment of Corona in a model” shows two unscaled orthogonal axes labelled “Time” and “Cases” with two “waves” starting in the origin, one high and short (in red), another flat and long (in green). A horizontal line marked “Capacity of the health care system” is tangent to the green “wave” at its peak. The figure text reads, “Without countermeasures the number of infected would increase rapidly and there would be an overload of hospitals”. An expert is cited, who calculated that “theoretically, around 2000 people with severe COVID-19 illnesses could be treated in intensive care units every day in Germany”.

Then the threat of overloading hospitals is explained: a continued similar speed of spread means the number of infected per day would raise to “several tens of thousands” and treating all in need of intensive care would be impossible. Therefore, it is important to “flatten the epidemiological curve”. “Scientists and politicians” call for avoiding social contacts as the only way to currently stop the rapid spread of the virus. There is also a link to an embedded report entitled “‘We have to save time.’ Chancellor Merkel emphasizes that the primary goal is to slow the spread of the virus”. In addition, the Spanish flu in 1918 is used to exemplify how social distancing has a “proven effect” with reference to a research article (via a link).

In this example of *rationalisation by explanation*, an attempt is made to explain the constructions used in depicting a looming crisis as a potential crisis of the health care system backed by the “theoretical” calculation of its capacity in general, and of German hospitals in particular. However, the numbers and the graph in this report are not treated as stemming directly from observation, but as *assumed* numbers in attempts of explaining some key metrics and mathematical notions used in the policy discourse. Also, the calculation of the capacities of German hospitals is labelled as “*theoretical*”. However, in order to achieve its effect, a link has to be made between the “theoretical” numbers and the particular policies introduced for “flattening the curve”. This is not achieved by any particular mathematical model, but by just placing side by side the “epidemiological curve” with the capacity in the image depicting two “waves”, although the theoretical possibility of using “more complex epidemiological calculations” that take countermeasures into account is mentioned. This is another strategy that we named *conjunction* (see description and further example below). Other examples of rationalisation by explanation include less “didactical” versions, mostly in the context of news items referring to official announcements of regulations or developments (Table 2: Nos. 17, 22).

#### Rationalisation by conjunction

We identified a strategy that involved juxtaposition of numbers from different fields, such as capacity of the health care system and epidemiology, or thresholds used for rules and regulations (e.g., events with 1000 people), which suggests a relation between these, although without any explanation or comparison of specific numbers. We see it as a form of rationalisation because, in addition to numbers, there are no other elements (such as examples used in *recharging*; see Sect. 4.2.5) to interrupt the expectation of objectivity. While the numbers can be recognised as elements of a scientific discourse, what exactly constitutes their relation or what these numbers have in common that points to the urgency of the proposed policy measures, remains implicit. This is in contrast to rationalisation by means of *explanation*, where an attempt of establishing these links is made. A prominent example we identified in a press conference with Chancellor Merkel.

- Example 2: *Merkel on the Corona crisis: discipline needed* (April 9)

The cover image shows Chancellor Merkel looking to the left at a German flag. In the middle of the page is a link to an embedded video of the press conference entitled “Angela Merkel on the current advice on the Corona pandemic.” We have analysed the event by using this link.

With the new numbers of infection decreasing, the Chancellor speaks of “a first success in the fight against the coronavirus”, appeals to adhere to the rules for contact restrictions during the Easter holidays as the situation is “fragile” and warns of a “false sense of security.” Even if the number of active infections goes down slightly, “when there are 200 people or more” who have lost their lives within one day, “these are simply numbers that cannot leave us cold” because “behind them stands in each case a human fate and a family, relatives and friends who mourn.” Also, when the “doubling times” have slowed down, or there is “improvement in the number of infections,” we still do not know “how an easing of our measures will work out.” The Chancellor also states, “We also saw how quickly the whole thing can grow exponentially and what it would have meant if it had continued like this” and paints a “worst” scenario of having to (re)introduce even stronger measures if “we were back in the range of exponential increase.”

In response to a question about using doubling time as a criterion for easing restrictions^5^, Merkel mentions “doubling time,” “reproduction number” and the “difference between the number of recovered and new cases” as different indicators of improvement, “the fact that the curve is no longer exponential but linear is one sign. There is a whole range of signs and the doubling time is one such indicator.”

With reference to health care resource statistics, the Federal Ministry of Health then states that the capacity of available intensive care beds in hospitals as well as the test capacity is more than sufficient. Also, the Federal Minister of Economics argues for a continued cautious course of action and compares continued discipline for a couple of more days with the even more problematic effect on economy, should the easing of measures have to be taken back.

The strategy of producing a *conjunction* of numbers here works by compiling of a range of metrics (as ”signs” of the virus) on the one hand, and numbers from public health care resource statistics on the other hand, to show the success of policy measures. There cannot be but a juxtaposition of particular numbers with the partial lockdown for suggesting the first being an effect of the latter, as there is no mathematised description of such a relation available. The numbers are then partly left to speak for themselves. The discourse also includes the imaginary of a worst scenario (a future of collective life “back in the range of exponential increase”) with the consequence of even more restrictive measures. The health crisis is constructed as overcome but lurking. Avoidance of an economic crisis is not in opposition with overcoming the health crisis, but subsumed under the same logic; more restrictive policy measures would worsen the economic situation even more. Other examples of the strategy include making links of numbers for regulations, such as upper limits of individuals allowed for events, with numbers from epidemiology (e.g., Table 2: No. 10) or juxtaposition of a range of numbers from epidemiology with the success of policy measures (Table 2: No. 28).

Overall, the policy for the management of the transition still appears as prescient rational decision making by the government based on the advice of weighty experts. However, we here also identified the strategy we termed *recharging* (see Sect. 4.2.5) of the numbers (“these numbers cannot leave us cold,” “behind them stands in each case a human fate and a family, relatives and friends who mourn”) to reinforce the appeals to observe the rules despite decreasing numbers.

#### Contrast

This strategy works by comparing numbers across contexts and periods of time or by comparing different metrics, all with the effect of establishing a stark contrast in terms of “high” and “low”; it so creates a tension between rationalisation and invoking fantasy by aggrandising differences between numbers through invoking emotions on one side of the comparison (e.g., a looming menace associated with high numbers). We identified it first in a text denying the evaluation of the situation as constituting a crisis in Germany (in contrast to numbers elsewhere); in consequence, the policy measures then should appear as questionable.

- Example 3: *Worry about the coronavirus: does Germany have to arm itself?* (January 22)

The theme of this report is the evaluation of the German situation by “experts.” There is an image showing the back of a person wearing an orange west with the label “health quarantine” holding an infrared thermometer (like a weapon). It is stated that in China “the number of infections with the new coronavirus is increasing rapidly,” including quotes and embedded links to interviews with three weighty experts. The notion of a “health risk for the population” is introduced with reference to an interview with the RKI-director; this risk is “rather low”; there are “low numbers of severely ill” in China of which “only a small part dies” with the conclusion that “the virulence or pathogenic potential of this virus can be assessed as small based on current knowledge.” In the other interviews, a possible scenario of “increased cases” is painted in the context of the preparation of hospitals for dealing with these. The interviewing journalist alerts us to “daily, or even hourly” rising reported cases of infections, but concludes that these appear to be still “manageable.” The report ends abruptly with: “Incidentally, influenza caused an estimated 25,000 deaths in Germany alone in the 2017/2018 season.”

Here all numbers are used to construct the epidemic in Germany as unproblematic: there is no crisis and no need for particular measures. This is achieved by establishing a contrast to China, by contrasting infected with severely ill or dying, and by (implicit) comparison of numbers with those (perhaps unexpectedly high) numbers related to influenza in Germany. As in Example 1, at the linguistic level, the interpretation of the numbers as a contrast is aided by using grammatical modifiers that qualify numbers in an absolute way, such as “rapid” increase contrasted with “only a small part.” In addition, in some passages we identified *association* (see Sect. 4.2.4) as a nested strategy. Further examples of *contrast* include comparison with numbers from China or Italy (Table 2: Nos. 1, 10, 21), or numbers associated with the flu (Table 2: No. 10), but also numbers of infected with numbers related to an upcoming crisis of “unimaginable scale” (Table 2: No. 18). This strategy is quite flexible in terms of supporting or opposing particular policies, as both the context of comparison and the selected numbers can be chosen freely.

#### Association

This label we have chosen for the juxtaposition of texts invoking numbers emanated from scientific discourse with representations of concrete content or manifestations of events, objects, operations, behaviour, etc. equivocal with overcoming the crisis or hindering this. The strategy often works with associating numbers with images, in particular where images may suggest either control or danger associated with the given numbers. In the following example the strategy is illustrated by an analysis of a video embedded in the news item.

- Example 4: *How the “R” comes about* (May 10)

The first theme of the news item is the importance of the reproduction number “R” as “critical value,” which according to the RKI is 1.10. “The RKI has repeatedly emphasized that in order to curb the epidemic, the reproduction number must be below one.” An embedded video is entitled “This is how the reproduction number is calculated.” There is an icon of a person having a white circle on the chest (the “virus”), the number “1” in a square, and a group of four persons, one of which also has a white circle on the chest. The video shows people walking in a city or sitting on a riverbank, including groups of young people without face masks talking to each other. A female voice comments by noting, ”Distance – here often far from it. Hamburg’s city centre full of people yesterday. Some of them with a mask but otherwise they stroll and chat like there is no Corona. A risky pastime.”

The video then shows a male person (labelled as physicist from the RKI) in casual clothes sitting in front of a laptop videotaping himself. The person explains, “From an epidemiological point of view, it can be expected that, of course, if we completely normalize the behaviour, we will come back to a scope where we have to expect increased numbers of cases.” Again, a short clip shows people walking in a pedestrian zone and young people tightly queuing in the background.

Then starts an animation with icons of persons and infected persons as on the opening image. The voice explains, “In this context the R-value, the reproduction number, is important.” It “indicates how many people on average are infected by an infected person.” The importance consists in “curbing the epidemic” and it should remain below one. The advantage of fewer people becoming infected is that they can be better “isolated and their contacts can be found”. As to the mathematical methods, the voice states the use of “now-casting, a complicated statistical procedure that is, however, quite imprecise.”

Thereafter the expert appears again, explaining, “You don’t get exactly one value, but an interval, for example between 0.8 and 1.3 and somewhere in there is the real value but you can’t measure it exactly.” It follows a similar clip as the previous one with people walking or sitting in a city, some without masks or in close contact. The scene is commented, “Because the scientists only know the reported cases they can only estimate how many people are actually infected. What is certain for them, however, is that without rules of conduct, such as keeping a distance, the virus will continue to spread”.

The major strategy of this video is *association* by juxtaposition of the explanation of a particular metric, the R-value, with the conduct of (mostly young) people shown in the video clips who obviously do not acquiesce. The relation of a specific value of the metric to the policy measures consists in stating that it should be below one, which is counteracted by the expert who asserts that there is only a range of values (e.g., “between 0.8 and 1.3”) and the “real value” cannot be measured. So there remains only association as a strategy. The only advice “scientists” can give (“what is certain for them”) consists in stating that without “rules of conduct” the virus will continue to spread. On the other hand, the general importance of numbers for what constitutes a rational policy discourse is stressed by providing technical background and explanation. A relation between the rules of conduct and the particular metric is posited (“in this context, the R-value is important”) and stressed by *association* of the potential further spread of the virus with a concrete manifestation of a behaviour affording this: the group of people with a “risky pastime” who increase the collective risk. The expert in the video, who counteracts the interpretation of the official current R-value of exactly 1.10 (by the RKI), which then would not have much meaning, appears as less prominent than the RKI-director.

Other examples of *associations* loosely linked numbers with appeals for observing the behaviour (Table 2: No. 18) or horrific social imaginaries (Table 2: Nos. 1, 18). Examples of associating numbers with images suggesting control include an image of a nurse wearing a protection dress and putting on her gloves (Table 2: No. 10), or of a person with a protection mask in a report about the “dark figure” of infections (Table 2: No. 40).

#### Recharging

This strategy consists in presenting concrete examples of people involved, first-hand experiences, personal narratives, testimonies, individual fates or other concrete illustrations of what can be found “behind” the metrics and so the numbers become *recharged* with subjectivity and materiality that has been eliminated through the mathematisation. For example, in establishing a routine based on a mathematisation using a particular calculation of doubling time together with a threshold as a criterion for easing restrictions (see example 3), the subject carrying the responsibility for decision making vanishes. In addition to example 2, above, where Chancellor Merkel used this strategy in her speech by explicitly alerting to the human fates “behind the numbers,” we found several examples of reporting personal stories of those who have died, as in the following example.

- Example 5: *China starts investigation after doctor’s death* (February 7)

This news item consists of a report and an embedded video about the death of Li Wenliang who worked at Wuhan Central Hospital. The opening image shows a photograph of the doctor surrounded by flowers. There is another photo of Li Wenliang in the text as well as a link to an embedded article entitled “The system made the crisis worse” with an image showing photographs of the President of the People’s Republic of China laid-out on a floor with feet in black boots stepping on them. The Wuhan Central Hospital is cited as reporting that all efforts to save the doctor had been in vain and, “We deeply regret it and mourn it.” Another theme concerns the collective reaction on social media in China. The subheadings read, “The fate symbolizes inaction by the authorities” and, “He couldn’t lie” as a quote by his mother who also says, “I and his father were healed. Unfortunately, my child did not survive.” Under the subheading “Doomed to silence“ mention is made of his warnings in an online discussion group also with reference to a possible return of the SARS-virus 17 years ago, which led to a pandemic with 8000 people infected and 774 dead. The story about the death of Li Wenliang is complemented by the information that there are more than 31,000 infections in China and that the National Health Commission has by then reported 636 deaths related to the coronavirus, in particular, the number of confirmed infections rose by 3143 to 31,161.

This example of *recharging* relies on reporting the individual fate of a significant person including passages in the style of an obituary amended by numbers of deaths and rise of infections. Other examples include reports on individual people affected in Germany in the beginning of the epidemic (Table 2: Nos. 3, 7, 8, 11) as well as about severely ill children in the USA (Table 2: No. 39).

In addition to the major discursive strategies described above, we found at the linguistic level the use of *(grammatical) modifiers* for single numbers (including mathematisations of the size of a difference between quantities) that qualify their size in an absolute way (examples 1 and 4), such as, “The still low fatality with 31 cases will change rapidly” (Table 2: No. 18), or qualifying the number of cases of infections after 2 months of exponential growth with a doubling time of 4 days as “more than 32 million“ (Table 2: No. 14) or “4 percent” as “high fatality rate” (Table 2: No. 32). A similar effect can also be realised by graphical modification (e.g., large size, bold print, red colour, large numbers with zeros rather than names), which we conjecture to be more common in the popular press (e.g., the examples given by Chassapis, 2017).

## Discussion

Our exploration unearthed four major discursive strategies embedded in a public discourse that heavily relies on numbers: rationalisation, contrast, association and recharging. In mathematics education the strategy of *rationalisation* has been associated with “dehumanized” bureaucratic rationality that excludes imagination and so is antithetical to social commitment (Ernest, 2010; Jablonka, 2020; Pimm, 1990; Walshaw, 2003). Even though we found the strategy of *rationalisation by explanation* that aided proceduralisation of decisions (e.g., by thresholds for measures), the numbers were not always presented as stemming from ready-made mathematical models developed by the experts; nonetheless this was envisaged when every now and then “more tangible” or “robust” numbers were promised to become available soon (Table 2: No. 32). Despite the massive use of numbers together with technical language, which altogether sets the standards for what counts as a rational policy discourse, the fragility of the constructions surfaced. We also identified a residual strategy of *rationalisation by conjunction* that creates the impression of fact, probability and verisimilitude only by means of repetition and compilation of numbers recognisable as elements of a scientific discourse. In parallel there were some black-boxes on offer, such as CovidSIM (2020) for simulating different epidemic scenarios or an interactive tool that calculates the individual risk of infection in different indoor environments (Dinklage et al., 2020). For the user, these may help to displace the lack of being in a position of attaining a definitive stance on what kind of inferences they can draw from “vague” numbers.^6^

On the other hand, we also identified strategies that invoked affective investment and fantasy by *contrast* (established by “large numbers” versus “small numbers” and associated scenarios), or by means of *association* of particular numbers with imaginaries of control or danger. The latter included indication of some specific manifestations of behaviour or events where people did not adhere to the measures. This association of numbers with an apparent concrete representation entails the possibility that the particular image is established as typical for a general category, as for example young people in close contact (example 4).^7^

*Recharging* emerged as another strategy that tries to get hold of the subject, which can be seen as combatting the “ethical filtration” (De Freitas, 2008; Skovsmose, 2006) of the rational-choice model of “collective risk” based on mathematical models of economic epidemiology and health care resource statistics (“flattening the curve”).^8^ The notion of ethical filtration alludes to the effect of reducing the complexity of handling a situation through its mathematisation, by which moral considerations or ethical dilemmas apparently are cleared away as the problem is transformed into a technical one.

The strategies of overcoming the apparent neutrality of depicting the problem by means of numbers appeared as an integral element of the public policy discourse, and so moralising the decision about both installing as well as complying to the rules and regulations formed part of it. In particular, the strategy of recharging was used in juxtaposition with rationalisation and not as its antipode.

While our investigation concerns a short-lived policy discourse that appeared relatively open and self-reflective in the context of a crisis, the strategies may be found in similar contexts where policies are directly related to reported numbers that define the changing scale of a problem that needs to be overcome. Important differences between contexts, which any further exploration would need to account for, concern the explicitness of the disciplinary function and the possibility of more than short-lived political or social identifications as for example in the case of environmental policy measures (cf. Hauge & Barwell, 2015). A more in-depth analysis might involve conjecturing about the workings of the strategies from affiliated or other theoretical vantage points.

As Ernest (2018) emphasises, mathematics education would be well advised to find means for eventually overcoming the training in “ethics-free” thought as the unintended outcome of an enculturation into school mathematical practice in which students usually do not engage in moral communication about particular mathematisations. In line with this invocation, Kennedy (2018, p. 310) argues for opening a philosophical space in the mathematics curriculum that “may have a place in the math classroom, helping to facilitate understandings about mathematics that may serve to complement and critically judge our inferences acquired in and with mathematics in the process of mathematization.” Our investigation contributes to the consolidation of these aims by a differentiation of discursive strategies of using numbers in public policy discourse. It offers a complement to epistemological reflections and so may assist in widening the repertoire of communications about mathematics in the curriculum.

In the larger context of research on mathematical literacy, the investigation can be read as a reminder of both the importance and the complexity of developing accounts of how numbers are used in public discourse. We consider such a recollection relevant, given that engaging with texts that contain numbers (ranging from news to instruction manuals) has become mainstreamed in competency-oriented conceptions of mathematical literacy via frameworks in international comparative tests and thereby attention to political dimensions has been truncated.

## Appendix

**Table 2:** Strategies for the use of numbers in 40 news items about the “Corona crisis” from the news outlet *tagesschau.de* during the observed period

Item no.*	1	2	3	4	5	6	7	8	9	10	11	12	13	14	15	16	17	18	19	20	21	22	23	24	25	26	27	28	29	30	31	32	33	34	35	36	37	38	39	40
Rationalisation: explanation		x	x			x	x	x			x	x	x	x	x	x	x		x	x		x		x		x		x	x	x	x	x			x	x		x		
Rationalisation: conjunction			x							x				x							x				x			x												x
Contrast	x					x			x	x								x	x		x											x								
Association	x				x													x				x	x	x	x	x	x						x	x	x		x			
Recharging			x	x			x	x			x														x								x						x	

*The entries are ordered chronologically (titles of the news items translated from German original by the authors): (1) Worry about the coronavirus—Does Germany have to arm itself? (Jan. 22); (2) Coronavirus in China: 56 deaths, almost 2000 infected (Jan. 26); (3) Meanwhile, 13 infected in Germany (Feb. 6); (4) China starts investigation after doctor’s death (Feb. 7); (5) More than 1000 deaths in China (Feb. 11); (6) Coronavirus in Germany—We need to manage the transition (Feb. 28); (7) Coronavirus in Germany: Infections in ten states (Mar. 2); (8) The new coronavirus: Cases increase, moderate risk (Mar. 3); (9) More than 100 new cases in Germany (Mar. 5); (10) “Spahn about the Coronavirus: Cancel events with more than 1000 people” (Mar. 8); (11) Two Corona-deaths in Germany (Mar. 9); (12) Denmark closes schools and day-care centres (Mar. 12); (13) Merkel about the Corona crisis: “abstain from social contacts when possible” (Mar. 12); (14) Coronavirus: Keep distance—slow down the virus (Mar. 14); (15) Corona crisis: Traffic restrictions in France (Mar. 15); (16) RKI grades risk as “high” (Mar. 17); (17) Bavaria announces restrictions for leaving home (Mar. 20); (18) RKI on the Corona crisis—crisis of “unimaginable scale” (Mar. 20); (19) How can the death rates differ? (Mar. 19); (20) Nationwide almost 5000 new infections (Mar. 26); (21) Nationwide almost 6000 new infections (Mar. 27); (22) RKI director Wieler on the coronavirus: The measures work (Apr. 3); (23) One million infected worldwide (Apr. 3); (24) Coronavirus—Experts question the Government policy (Apr. 6); (25) Merkel on the Corona crisis: Discipline needed (Apr. 9); (26) RKI on the Corona situation: We can see a slowdown (Apr. 14); (27) RKI director Wieler: “The control strategy shows success” (Apr. 17); (28) Excess mortality: 100.000 more deaths within four weeks (Apr. 27); (29) Reproduction number: Measuring the pandemic (Apr. 28); (30) RKI on the Corona pandemic: Increased infection rate in Germany (Apr. 28); (31) Infection rate again 0,9 (Apr. 28); (32) Corona numbers from RKI - A warning despite fewer new infections (Apr. 30); (33) More than 10.000 new cases in Russia (May 3); (34) 32000 Corona deaths: Sorrowful record in Great Britain (May 5); (35) Report with embedded video: How the “R” comes about (May 10); (36) Reproduction number exceeds critical value (May 10); (37) Even more Corona cases in Coesfeld (May 11); (38) Metric for epidemics: What is the R-value? (May 12); (39) “It was said that children should not be affected” (May14); (40) Health care professions and Corona: Helpers at risk (May19). On September 1, 2020, these news items were available at https://www.tagesschau.de/archiv/meldungsarchiv100.html

## Footnotes

1 Neither do we want to suggest that for this reason moralising the use of mathematical models in the context of the pandemic automatically becomes productive (cf. Lensing & Straehler-Pohl, 2018).

2 By March, the shortened form “Corona-Krise” (Corona crisis) appeared after longer or less specific forms (e.g., “the virus crisis in China” or “the coronavirus crisis”) in German news. *Corona* was used as metonym for the virus, for the disease or even the social problems caused by it.

3 During the second and the third wave of the pandemic in Germany (starting October 2020) there was a focus on *7-day incidence*, *reproduction number* and *intensive care capacity*, by which benchmarks were set for introducing, maintaining or easing measures for curbing the pandemic by the federal and local governments. Absolute numbers for daily cases and deaths also remained central in the public discourse.

4 Detailed descriptions are available in the daily epidemiological reports published by the RKI (RKI, n.d.).

5 Using a threshold of a doubling time of 10 days (without specifying the reference period for the calculation) had been suggested by the Chancellor earlier.

6 Straehler-Pohl’s (2015) account of the “ideology of certainty” opens up a productive route for theorising this observation.

7 Similarly, Warren (2011) noticed in official statistics reports on human trafficking photo collages picturing young women as typical victims complementing the numbers.

8 A similar observation has also been made by Warren (2011) who found the use of narrative portrayals and testimonies of victimization in his analysis of statistics reports.
